# Oxidized cholesterol species as signaling molecules in the brain: diabetes and Alzheimer’s disease

**DOI:** 10.1042/NS20190068

**Published:** 2019-11-28

**Authors:** Thaddeus K. Weigel, Joshua A. Kulas, Heather A. Ferris

**Affiliations:** 1 Department of Neuroscience, University of Virginia, Charlottesville, VA 22908, U.S.A.; 2 Division of Endocrinology and Metabolism, University of Virginia, Charlottesville, VA 22908, U.S.A.

**Keywords:** Alzheimers disease, cholesterol, oxysterols, type 2 diabetes

## Abstract

Type 2 diabetes is associated with adverse central nervous system effects, including a doubled risk for Alzheimer’s disease (AD) and increased risk of cognitive impairment, but the mechanisms connecting diabetes to cognitive decline and dementia are unknown. One possible link between these diseases may be the associated alterations to cholesterol oxidation and metabolism in the brain. We will survey evidence demonstrating alterations to oxysterols in the brain in AD and diabetes and how these oxysterols could contribute to pathology, as well as identifying research questions that have not yet been addressed to allow for a fuller understanding of the role of oxysterols in AD and diabetes.

## Introduction

Type 2 diabetes is a driver of Alzheimer’s disease (AD) symptomatology [1] and cognitive decline [2,3], likely through multiple mechanisms that result in a doubled risk of AD [1]. Diabetes and AD share several common brain phenotypes that have been proposed as possible links between the diseases. AD and diabetes both increase insulin resistance, inflammation, and oxidative stress in the brain [4,5]. Vascular disease associated with diabetes increases risk of dementia [6,7], and both hypoglycemia [8] and hyperglycemia [9,10] may also contribute to AD risk. Diabetes and AD have also both been linked to disruptions in cholesterol metabolism in the CNS [11–13]. The brain is highly cholesterol dense, and cholesterol is essential for neuronal functions such as synapse formation, vesicle fusion, and membrane receptor clustering [13–15]. Genetic evidence strongly implicates cholesterol in AD. The largest known genetic risk factor for late onset AD, the most common form of the disease, is the ϵ4 allele of apolipoprotein E (ApoE) [16]. ApoE is a lipid-binding protein that forms high-density lipoprotein (HDL)-like particles to transport cholesterol and other lipids throughout the brain. Other risk genes for AD are also associated with cholesterol, including ApoJ/CLU (which interacts with ApoE to form cholesterol-carrying lipoproteins) [17–19], ABCA7 (a membrane cholesterol transporter) [17,20], and SORL1 (an ApoE receptor involved in internalizing cholesterol-carrying lipoproteins) [17,18]. Studies of rodent models of diabetes have shown decreased cholesterol synthesis in the brain [12]. Direct modulation of cholesterol synthesis in the brain has been proposed as a possible therapeutic strategy in AD, and cholesterol synthesis inhibitors can decrease amyloid β and tau phosphorylation *in vitro* [21,22]. Observational studies in humans have reported that use of the cholesterol synthesis-impairing statins is correlated with decreased AD risk [23,24], but randomized placebo-controlled trials have not found statins to decrease AD incidence [25–27]. Other than through alterations in biosynthesis, cholesterol metabolism could affect brain health through changes in oxidized cholesterol species, known as oxysterols. Levels of these molecules are altered in AD and diabetes, and may have broad effects on important mechanisms in neurodegeneration including neuroinflammation, CNS cholesterol regulation, and neuronal health and survival.

## Oxysterols

**Figure 1:**
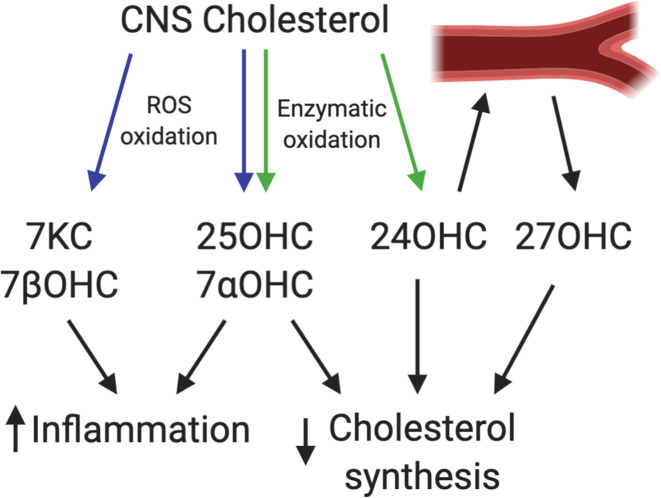
Oxysterols in the brain Oxysterols are produced from cholesterol by autoxidation or enzymatic oxidation. Non-enzymatically produced oxysterols include 7-ketocholesterol (7KC) and 7β-hydroxycholesterol (7βOHC). Other oxysterols are produced predominantly enzymatically, including 24(S)-hydroxycholesterol (24(S)OHC), which is produced exclusively in the brain for cholesterol export to the blood. 7α-Hydroxycholesterol (7αOHC) and 25-hydroxycholesterol (25OHC) are produced both enzymatically and non-enzymatically. 27-Hydroxycholesterol (27OHC) is produced in the periphery and enters the brain through the blood. These oxysterols can contribute to neuroinflammation and decreased cholesterol synthesis, two mechanisms thought to contribute to neurodegeneration.

Cholesterol can be oxidized at various positions and by enzymatic or non-enzymatic mechanisms, resulting in a variety of structurally and functionally distinct oxysterols. The 7-carbon of cholesterol is particularly vulnerable to autoxidation, yielding oxysterols including 7-ketocholesterol (7KC) and 7β-hydroxycholesterol (7βOHC). Thus 7KC and 7βOHC production is a function of ROS levels in cells (Figure 1). Other oxysterols are produced enzymatically, primarily by members of the cytochrome P450 family: 7α-hydroxycholesterol (7αOHC) is produced by CYP7A1, 25-hydroxycholesterol (25OHC) by cholesterol 25-hydroxylase (CH25H), 24(S)-hydroxycholesterol (24(S)OHC) by CYP46A1, and 27-hydroxycholesterol (27OHC) by CYP27A1. In addition to their enzymatic production, 7αOHC and 25OHC can also be generated non-enzymatically.

## Oxysterol production and regulation in the brain

The oxysterol content of the brain differs from that of the blood and other peripheral tissues because of CNS-specific patterns of lipid composition and oxysterol generation and export. The blood–brain barrier (BBB) is impermeable to cholesterol, meaning that cholesterol in the brain must be synthesized *in situ*. In the adult brain this is performed primarily by astrocytes, which utilize some of that cholesterol and export the rest for use by neurons and other glia. This cholesterol can subsequently be oxidized, either to serve as a signaling molecule, to facilitate cholesterol clearance from the CNS or as a consequence of oxidative stress. Unlike cholesterol, these less hydrophobic oxysterols can be cleared from the brain by crossing the BBB, with subsequent transport via the blood to the liver. Permeability of the BBB to oxysterols means that they can also cross from the blood into the brain. In humans the flux of the oxysterols 24(S)OHC, 7βOHC, and 7KC is out of the brain [28], while flux of 27OHC is into the brain [29].

Enzymatic cholesterol oxidation differs in the brain compared with other tissues. 24(S)OHC is a CNS-specific oxysterol that is essential for regulating brain cholesterol content. Because cholesterol cannot cross the BBB and is not broken down locally, excess in the brain is converted into the BBB-permeable oxysterol 24(S)OHC by CYP46A1 expressed in neurons. 24(S)OHC is then exported to the blood where it is carried by LDL to the liver for degradation and excretion. Because it has such an important role in cholesterol clearance and not just as a signaling molecule, 24(S)OHC is the most prevalent oxysterol in the brain. Regulation of CYP46A1 is not well understood, and at the transcriptional level it is not altered by cholesterol, oxysterols, or statin treatment [30]. Its activity can be increased *in vitro* by direct interactions with neurotransmitters, particularly glutamate [31], and several drugs have been found to modulate CYP46A1 activity [32–34]. The antiretroviral efavirenz increases CYP46A1 activity *in vitro* and increases 24(S)OHC levels *in vivo* [33], while the antifungal voriconazole inhibits CYP46A1 and decreases 24(S)OHC production *in vivo* [32]. These treatments induce broader alterations to brain cholesterol metabolism, with the CYP46A1 antagonist causing decreased cholesterol synthesis in the brain and CYP46A1 agonist inducing increased brain cholesterol synthesis. In both cases, this results in unchanged total brain cholesterol levels. That a CYP46A1 agonist would increase cholesterol synthesis may seem paradoxical, as 24(S)OHC is a negative regulator of the transcription factor promoting cholesterol synthesis [35]. However, cholesterol is also an inhibitor of cholesterol synthesis through this same system [36], and increased activity of CYP46A1 will decrease cholesterol levels and will relieve this inhibition of cholesterol synthesis. This demonstrates a complex mechanism in the brain that senses and responds to changes in cholesterol levels and oxidation to maintain cholesterol homeostasis in the CNS. CYP46A1 inhibition induces astrocyte activation in the retina [37], suggesting that in addition to synthesizing the bulk of the cholesterol in the brain, astrocytes may also be responsible for detecting changes in brain cholesterol metabolism and compensating for them. The role that CYP46A1 and 24(S)OHC plays in the brain, facilitating the clearance of excess cholesterol by the blood to the liver, is performed in other tissues by CYP27A1 and its oxysterol product 27OHC. 27OHC levels are higher in the blood than in the brain, resulting in a flux of 27OHC into the brain from the periphery [29]. Though this oxysterol is not primarily derived from the CNS, it may play important roles in health and pathology in the brain, discussed below.

25OHC, another important oxysterol in the brain, is produced by CH25H. It is not necessary for cholesterol export from the brain and instead serves primarily as a signaling molecule. 25OHC is best studied in immune function, and CH25H activity is induced in macrophages by treatment with LPS or the cytokines IFN-α, IFN-β, and IFN-γ [38,39]. 25OHC acts at multiple levels of the immune response: it not only acts as a signaling molecule to amplify inflammatory activation in macrophages [40], but also has direct antimicrobial effects, impairing virus entry and replication [41,42]. The role of 25OHC in the brain is poorly understood. 25OHC is elevated in the brain in X-linked adrenoleukodystrophy, where it promotes microglial activation and oligodendrocyte death through NLRP3 inflammasome activation [43]. The roles that 25OHC may play in the healthy brain need to be further studied.

In addition to tissue-specific patterns of enzymatic cholesterol oxidation, non-enzymatic oxysterol production plays important roles in the brain. The brain is particularly susceptible to non-enzymatic cholesterol oxidation because of the high concentration of cholesterol combined with high oxygen consumption and ROS generation in the CNS. Unlike enzymatically generated oxysterols, production of these species cannot be regulated at the transcriptional or enzymatic level. The rate of production of non-enzymatically generated oxysterols in the brain varies as a function of the balance between ROS generation and antioxidant function, with more oxysterols produced when ROS generation outstrips the cell’s ability to reduce reactive species before they cause oxidative damage to lipids. While non-enzymatic oxysterol production cannot be directly regulated, levels of these oxysterols can be regulated through mechanisms of clearance. ATP-binding cassette (ABC) efflux pumps play important roles in exporting oxysterols from cells in the brain, and the expression of these transporters is one mechanism by which intracellular oxysterol levels can be regulated. In fact, different oxysterols can regulate each other by modulating efflux pump expression. As an example, 24(S)OHC treatment induces expression of ABCG1 through activation of the liver X receptor (LXR). Cultured neurons pre-treated with 24(S)OHC have increased resistance to 7KC-induced apoptosis through an increase in ABCG1-mediated 7KC efflux [44].

Because cholesterol and oxysterols are not broken down in the brain, clearance of these molecules requires their export across the BBB and into the blood so that they can be transported to the liver. This export process is still not fully understood, but it is thought to be driven by passive diffusion and active transporters in the neurons and glia and at the endothelial cells of the BBB. Sidechain-oxidized oxysterols (such as 24(S)OHC, 27OHC, and 25OHC) are particularly capable of being expelled from lipid membranes, allowing them to diffuse across plasma membranes and into the blood [45]. Active transport may also play a role in export of oxysterols across the BBB, as an inhibitor of organic anion transporter proteins (OATPs) decreases efflux of 24(S)OHC from the brain in rats [46].

## Oxysterols in AD and diabetes

Oxidative stress is an important mechanism in AD. AD is characterized by both increased ROS production and decreased antioxidant function in the CNS. Treatment of neurons, astrocytes, and microglia with amyloid β increases ROS generation *in vitro* [47–50], and fatty acid peroxidation, a result of oxidative damage to membranes, is increased in AD brains [51,52]. Meanwhile the important antioxidant polypeptide glutathione (GSH) is decreased in the brain in AD [53], and the severity of cognitive decline in AD is correlated with decreased GSH levels in the hippocampus and frontal cortex [54]. As cholesterol autoxidation is a function of ROS levels, this increased ROS generation and decreased antioxidant function in AD results in increased generation of non-enzymatically produced oxysterols. Levels of the ROS-generated oxysterols 7KC and 7βOHC are elevated in cerebral cortical tissue from AD patients [55]. 7KC is also elevated in the blood in AD [56], likely as a result of the ability of oxysterols to cross the BBB into the periphery.

Oxidative stress is also observed in diabetes, though it is far less well studied in the brain than in AD. Fatty acid peroxidation resulting from oxidative damage to lipid membranes is increased in the brain in Zucker diabetic rats [57], a genetic model of Type 2 diabetes, and in rats treated with streptozotocin (STZ) [58], a model of Type 1 diabetes. Studies of high-fat diet (HFD), which can induce symptoms of metabolic syndrome in rodents, have examined oxidative stress in the brain more extensively. HFD increases ROS and oxidative damage while decreasing GSH levels and the activity of superoxide dismutase (SOD), an antioxidant protein, in the hypothalamus in rats [59]. HFD also elevates oxidative stress and decreases antioxidant function in the hippocampus in mice, and these effects can be rescued by melatonin, which can act as an antioxidant [60]. The increased oxidative stress observed in these models suggests that non-enzymatically produced oxysterols would be elevated in the brain in diabetes similar to what is seen in AD. The only study directly measuring oxysterols in the brain in diabetes models found that rats treated with STZ have elevated 7KC and 7βOHC in the cortex [61,62]. While oxysterols in the brain are poorly studied in diabetes, oxysterols have been found to be elevated in the blood of diabetes patients [63] and are correlated with HbA1c levels, a measure of glucose control [64]. 7βOHC is also elevated in the blood in diabetes patients [65]. Oxysterols in the brains of diabetes patients still have not been measured, but these findings from studies of rodent brains and human blood suggest that, like AD, diabetes probably results in increased levels of non-enzymatically produced oxysterols in the brain.

Enzymatically produced oxysterols are also affected by AD and diabetes, though some of these results are still disputed. 24(S)OHC has been reported to be both increased [66] and decreased [55,67,68] in the brain and CSF of AD patients. Some of these conflicting results may come from differences in disease stage being studied, where 24(S)OHC may be elevated in early AD but decrease when neurodegeneration progresses and the neurons that express CYP46A1 begin to die, but more research on this topic is necessary. Though it has not been studied in the brain in human diabetes, 24(S)OHC is decreased in the brain of a rodent diabetes model [61]. 27OHC, which is derived from the periphery but can cross the BBB, is elevated in AD brains [55,69]. Again, data on oxysterols in the brain in diabetes are limited, but enzymatically produced oxysterols are likely altered in this disorder as well. STZ-treated insulin-deficient rats have decreased 24(S)OHC and 27OHC in the cerebral cortex [61]. In humans, 27OHC and 25OHC are elevated in the blood in patients with Type 2 diabetes compared with controls [70]. These differing results regarding 27OHC may be attributable to the importantly distinct effects on peripheral metabolism from the obesity and insulin resistance of Type 2 diabetes versus the rodent model of lean, insulin-deficient Type 1 diabetes. The peripheral metabolic effects of Type 2 diabetes are likely responsible for alterations in both blood and brain 27OHC levels, as it is produced in the periphery and then diffuses into the brain, but these changes may have substantial signaling effects in the brain.

## Oxysterol toxicity and signaling functions

Both enzymatically and non-enzymatically produced oxysterols have broad signaling functions involving a number of pathways that are altered in diabetes and AD. Here we will discuss CNS cholesterol regulation, oxidative stress, and inflammation.

Perhaps the most important role for oxysterols in the brain is in regulating cholesterol synthesis and transport. Numerous genes identified as risk factors for AD are related to cholesterol transport and metabolism in the brain, including ApoE [16], ApoJ/CLU [17–19], ABCA7 [17,20], and SORL1 [17,18]. Cholesterol synthesis in the brain is also decreased in multiple mouse models of diabetes [12]. Brain cholesterol metabolism is altered in aging: cholesterol synthesis is decreased [71,72] and cortical cholesterol content is decreased [73], though this decreased cholesterol level has not been observed in the hippocampus [71]. Cholesterol synthesis is driven by the transcription factor sterol regulatory element-binding protein-2 (SREBP2), which controls transcription of the genes involved in the cholesterol synthetic pathway. SREBP2 resides in the ER, bound to the proteins SCAP and Insig, which retain SREBP2 in the ER. Cholesterol and oxysterols facilitate the interaction between SCAP and Insig that retain SREBP2 in the ER. In sterol-poor conditions 25OHC levels decline, destabilizing the SCAP–Insig interaction and allowing SREBP2 to be transported to the Golgi. There it undergoes proteolytic cleavage to release its transcriptionally active N terminus, which travels to the nucleus and drives transcription of cholesterol synthetic genes. Oxysterols, particularly 24(S)OHC and 25OHC, are repressors of the transport of SREBP2 to the Golgi and subsequent induction of cholesterol synthesis [35].

Oxysterols can contribute to both oxidative stress and antioxidant defense. Several oxysterols, most notably 7KC, are known to induce ROS generation [74,75]. Treatment with high concentrations of 7KC induces apoptosis in cultured macrophages in an oxidative stress-dependent mechanism [76]. Because 7KC is both a product and an inducer of ROS generation, it may contribute to a positive feedback loop resulting in increased oxidative stress in the brain. On the other hand, other oxysterols can counteract increased oxidative stress. 24(S)OHC can activate sirtuin 1 (SIRT1), a histone deacetylase protein involved in regulating transcription of important antioxidant genes [77].

Oxysterols can additionally act as potent immune signaling molecules. 7KC in particular has been studied in the context of inflammation. Neuroinflammation is an important component of AD pathology and is thought to play a mechanistic role in neurodegeneration. Peripheral immune activation has long been identified in diabetes, but more recent research has demonstrated neuroinflammation in response to high-fat diet [78–81]. 7KC activates the immune sensor toll-like receptor 4 (TLR4) [82] and can induce the secretion of pro-inflammatory cytokines from macrophages and microglia. In the brain, this may contribute to neuroinflammation, gliosis, and increased risk for neurodegeneration.

## Microglia and oxysterols in disease

Microglia are the brain’s resident immune cells and play an active role in surveying the brain and responding to a variety of insults. Unlike other tissue-specific macrophages, which originate from bone marrow progenitor cells, microglia derive from the yolk sack and migrate to the brain during development [83]. Microglia are motile cells that extend numerous branched processes from their cell bodies and are well-known to undergo morphological changes in response to pro-inflammatory stimuli. Like peripheral macrophages, microglia are capable of phagocytosing pathogens and cellular debris to protect the brain, and are thought to play an important role in regulating synaptic connections [84].

Abundant evidence has linked microglia to neurodegenerative disease. Microglial activation is a widely observed phenomenon in mouse models of AD and in human AD [85]. Mutations in Trem2, a cell surface protein found on microglia, are a risk factor for AD [86,87]. Pro-inflammatory shifts in microglial phenotype have also been observed in response to HFD and in mouse models of diabetes [88]. Recent research has shown that pharmacologic depletion of microglia from the brains of mice maintained on a HFD reduces food intake and weight gain [89]. However, depleting microglia in mouse models of AD can surprisingly result in a loss of parenchymal amyloid plaques [90,91]. Thus, microglia appear to play a complex role in the brain response to disease and are capable of influencing the progression of brain diseases.

It is well characterized that peripheral immune cells phagocytose oxidized cholesterol laden lipoproteins. When cholesterol levels are elevated in the periphery, macrophages consume excess oxidized cholesterol molecules and shift to an inflammatory phenotype [92]. While macrophage sequestration of oxysterols is likely protective, eventually, buildup of these cholesterol-laden immune cells in blood vessel walls is associated with vascular damage and ultimately atherosclerosis. Compared with macrophage biology, much less is known about how oxysterols, including 7KC, impact microglia. Early work by Chang et al. demonstrated that some oxysterols, including 25OHC, may act directly on the N9 microglial cell line, potently stimulating induction of iNOS, increasing the inflammatory state of the cells and inducing toxicity [93]. They subsequently demonstrated that treatment of N9 cells with micromolar concentrations of 25OHC potently induces c-Jun signaling and that this effect could be inhibited by treatment with PPAR receptor agonists [94]. This research helped lay the groundwork establishing that oxysterols act as pro-inflammatory inducers in microglial cells *in vitro*.

Several groups have contributed to the understanding of how oxysterols drive microglial activation in disease models and *in vivo*. A large fraction of the brain’s cholesterol content is found in myelin. Demyelinating diseases, such as multiple sclerosis, significantly increase 7KC levels in CSF, likely due to myelin breakdown [95]. 7KC itself, in turn, is a robust inflammatory activator of the BV2 microglial cell line, suggesting that 7KC may be a signal to microglia for debris engulfment. 7KC induces iNOS expression and promotes nuclear accumulation of the pro-inflammatory transcription factor NFκB. Poly ADP-ribose polymerase 1 (PARP-1) modifies proteins post-translationally via conjugation of ADP-ribose moieties, a process that has been shown to play an important role in immune cell differentiation and adaptation to extracellular danger signals [96]. Interestingly, siRNA targeting of PARP-1 is sufficient to block the pro-inflammatory and toxic effects of 7KC in cultured BV2 cells and in brain tissue/microglia co-cultures. This work suggests that, in addition to 7KC being elevated in demyelinating diseases and AD, it is sufficient to promote neurotoxicity. Moreover, the neurotoxic effects may be dependent on microglia. This concept of PARP-1 playing an essential role in oxysterol induced inflammation was further demonstrated in studies examining 15α-hydroxicholestene (15HC), another oxidized cholesterol metabolite that is significantly increased in the serum of MS patients. 15HC stimulated a robust increase in iNOS and TNFα in primary microglial cultures and robustly activated PARP-1 enzyme activity [97]. The oxysterol induced PARP-1 is dependent on the pattern recognition receptor TLR2. This suggests that some oxysterols promote their inflammatory effects by activating cell microglial pattern recognition receptors in the brain to induce a neuroinflammatory cascade. In addition to TLR2, TLR4 can also act as a receptor for 7KC [82]. Interestingly, TLR-driven activation of PARP-1 has also been implicated as a mechanism for Aβ microglia activation, suggesting that oxysterols and Aβ may synergistically stimulate microglia through a common signaling pathway [98,99].

The retina can often provide significant insights into the biology of the brain. Oxysterols may play a particularly important role in the retina. 7KC content increases in the mouse retina with age [100]. It has also been implicated in diabetic retinopathy. Either as part of an oxidized LDL complex or on its own, 7KC can induce apoptosis of pericytes [78]. 7KC stimulates retinal microglia, changing their morphology and causing increased expression of pro-inflammatory cytokines. Moreover, LPS-primed retinal microglia were found to have increased inflammasome activation by increased NRLP3 induction in response to 7KC. This resulted in significantly increased secretion of cytokines including TNFα, IL-6, and IL1β. 7KC can also lead to the down-regulation of neurotrophic factor expression in microglia, including brain-derived neurotrophic factor (BDNF), suggesting that elevated levels of 7KC may promote inflammation and disrupt important factors in maintaining neuronal survival and function. These findings were consistent with work that demonstrated that 7KC inflammasome induction also occurs in human microglia [101].

While the pro-inflammatory effects of oxysterols on microglia have been consistently observed, how inflammatory signals in the brain affect the production of oxysterols in cells is poorly understood. In a recent study, BV2 microglial cells were treated with LPS and several oxysterols were measured over 24 h [102]. Non-enzymatically produced 7KC was significantly increased, while several enzymatically produced oxysterols, including 25OHC and 27OHC, were down-regulated. This is consistent with the notion that LPS stimulation will cause increased ROS production, causing increased non-enzymatic cholesterol oxidation in turn. LPS also drives changes in the expression of cholesterol oxidizing cytochrome P450 enzymes, increasing the cholesterol synthetic pathway CYP7B1 expression, while reducing CYP27A1 and CYP46A1 expression. In addition to LPS driving oxysterol profile changes, interleukin-4 is also capable of changing oxysterol levels. Thus, it appears that oxysterol production is dynamically regulated in microglia in response to environmental signals.

**Figure 2:**
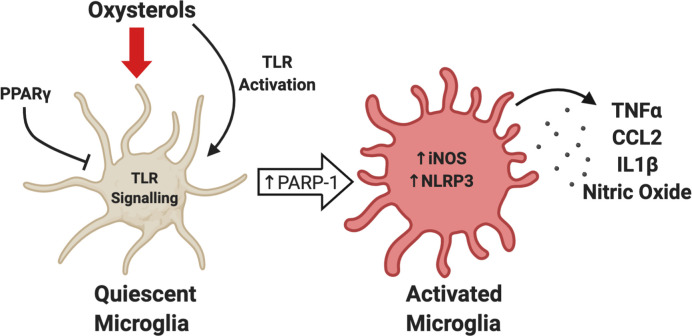
Microglia activation by oxysterols Oxysterols, including 7-ketocholesterol, stimulate microglia by activating toll-like receptors (TLRs). TLR activation drives an intracellular signaling cascade dependent on kinases including Akt, P38, PKC, PI3K, and ERK1/2 [97]. TLR signaling activates PARP-1 enzyme activity and ultimately shifts microglia to a pro-inflammatory activated state. Oxysterol activation of microglia can be prevented by treatment with PPARγ receptor agonists. Oxysterol activated microglia have activated inflammasome markers including increased NLRP3 and caspase 1. iNOS expression is also robustly up-regulated. Activated microglia then secrete a variety of immune signaling molecules including TNFα, CCL2, IL1β,nd nitric oxide.

Taken together these studies demonstrate a clear role for oxysterols, and particularly 7KC, as potent pro-inflammatory molecules that are sufficient to activate microglia. The effect of oxysterols on microglia is modeled in Figure 2. Oxysterol levels have been shown to increase in metabolic disease and in AD (reviewed above); thus, increased oxysterol levels may play an important role in these diseases by promoting microglial mediated inflammation to exacerbate the neurodegenerative process.

## Conclusion

Oxysterol levels are altered in AD and diabetes, reflecting changes in both oxidative stress and enzymatic regulation of cholesterol oxidation. These oxysterols are potent signaling molecules and may contribute to mechanisms of neurodegeneration. 7KC in particular is elevated in AD and diabetes, is an inflammatory signaling molecule, and can induce immune activation in microglia. Enzymatically produced oxysterols such as 25OHC and 24(S)OHC, known modulators of cholesterol metabolism, are also altered in AD and diabetes. Increased neuroinflammation and decreased cholesterol synthesis are possible mechanisms in the progression of neurodegeneration and changes in oxysterols in the brain may contribute to them both. Still, many questions remain to be answered. The effects of diabetes on brain oxysterol levels are understudied. The cell-type specific effects of oxysterols in the brain are also mostly unexamined. The pro-inflammatory effects of 7KC and other oxysterols on microglia are discussed here, but oxysterols likely have variable and important effects on different cells. Astrocytes are the main cholesterol producers of the brain, oligodendrocytes contain dense cholesterol deposits in their myelin, and neurons depend on cholesterol in the dynamic membranes at their synapses; oxysterols could have distinct effects on all these cells in neurodegeneration. Further research will give a clearer picture of the mechanistic roles that oxysterols may play linking diabetes and AD.

## Abbreviations

15HC: 15α-hydroxicholestene
AD: Alzheimer’s disease
ApoE: apolipoprotein E
BBB: blood–brain barrier
BDNF: brain-derived neurotrophic factor
HDL: high-density lipoprotein
HFD: high-fat diet
OATP: organic anion transporter protein
PARP-1: Poly ADP-ribose polymerase 1
SIRT1: sirtuin 1
SOD: superoxide dismutase
SREBP2: sterol regulatory element-binding protein-2
TLR: toll-like receptor
